# Coagulopathy as a Prodrome of Cytokine Storm in COVID-19-Infected Patients

**DOI:** 10.3389/fmed.2020.572989

**Published:** 2020-10-23

**Authors:** Hui Guo, Ying Sheng, Wei Li, Fei Li, Zongyu Xie, Jing Li, Yuhe Zhu, Jian Geng, Gang Liu, LeJian Wang, Jing Li, Fengchao Wang

**Affiliations:** ^1^Department of Laboratory Medicine, The First Affiliated Hospital of Bengbu Medical College, Bengbu, China; ^2^School of Nursing, Indiana University, Indianapolis, IN, United States; ^3^Department of Respiratory Disease, The First Affiliated Hospital of Bengbu Medical College, Bengbu, China; ^4^Department of Laboratory Medicine, Taizhou Central Hospital (Taizhou University Hospital), Taizhou, China; ^5^Department of Radiology, The First Affiliated Hospital of Bengbu Medical College, Bengbu, China; ^6^Department of Laboratory Medicine, Bengbu Medical College, Bengbu, China; ^7^Department of Thoracic Surgery, The Second Affiliated Hospital of Bengbu Medical College, Bengbu, China; ^8^Department of Laboratory Medicine, Zhejiang University of Traditional Chinese Medicine Affiliated XinHua Hospital, Hangzhou, China; ^9^Department of Surgery, University of Michigan School of Medicine, Ann Arbor, MI, United States

**Keywords:** COVID-19, coagulopathy, cytokine storm, prodrome, d-dimer, IL-6

## Abstract

**Background:** The rapid coronavirus disease 2019 (COVID-19) pandemic has hit hard on the world and causes panic since the virus causes serious infectious respiratory illness and easily leads to severe conditions such as immune system overactivation or cytokine storm. Due to the limited knowledge on the course of infection of this coronavirus and the lack of an effective treatment for this fatal disease, mortality remains high. The emergence of a cytokine storm in patients with a severe condition has been reported as the top reason of the death of patients with COVID-19 infection. However, the causative mechanism of cytokine storm remains elusive. Thus, we aim to observe the association of coagulopathy (D-dimer) with cytokine (i.e., IL-6) and CT imaging in COVID-19-infected patients.

**Methods:** In this retrospective observational study, we systematically analyzed the comprehensive clinical laboratory data of COVID-19-positive patients in different illness groups of mild, moderate, and severe conditions according to the Chinese Clinical Guidance for COVID-19 Pneumonia Diagnosis and Treatment (7th edition). *T* tests and chi-square tests were used for two-group comparisons. One-way ANOVA was used for three-group comparisons. Pearson and Spearman correlation coefficients of the D-dimer level with IL-6 and CT imaging were computed at baseline. With regular liquid biopsy approach, D-dimer, IL-6, and neutrophil-to-lymphocyte ratio were recorded repeatedly with a time curve to investigate disease progression, along with CT imaging, and other indicators.

**Results:** All the 64 patients were clinically evaluated and classified into three groups of mild (32 cases), moderate (23 cases), and severe (nine cases) conditions. The D-dimer level positively correlated with IL-6 (*R* = 0.5) at baseline when the COVID-19-infected patients were admitted. In addition, we observed that D-dimer rises earlier than the cytokine storm represented by IL-6 surge, which suggests that coagulopathy might act as a trigger to potentiate a cytokine storm.

**Conclusion:** Integrated analysis revealed a positive correlation of coagulopathy with cytokine storm in COVID-19-infected patients; the D-dimer rises early, which indicates that coagulopathy acts as a prodrome of cytokine storm. Coagulopathy can be used to monitor early cytokine storm in COVID-19-infected patients.

## Introduction

COVID-19 has become a global pandemic. The coronavirus is a large virus family, which was known to cause serious infectious respiratory illnesses such as Middle East respiratory syndrome, severe acute respiratory syndrome (SARS), and SARS-CoV-2 (1–5). SARS-CoV-2 infects humans via the same receptor as SARS-CoV—human angiotensin converting enzyme II (6). Coronavirus disease 2019 (COVID-19) is a disease widely spread across continents and oceans, which causes severe damages to the human body and panic in the world. Due to the limited knowledge on the course of infection of this coronavirus, the mortality of COVID-19-infected patients remains high (7, 8). Cytokine storm was observed and is considered as the top reason of death in COVID-19-infected patients (9–11). The unexpected emergence of a cytokine surge frequently appears in patients with severe conditions of pneumonia (11–13). However, the causative origin of a cytokine storm is unknown. The elusive trigger of a cytokine storm renders effective prevention and treatment impossible. Therefore, we used COVID-19 infected patients as a model to study the origin of a cytokine storm. Identifying the origin of a cytokine storm will enable us to act early to block or decelerate its lethal progression.

Cytokine storm starts locally in the lung and is abruptly activated in the systemic-level circulation, which results in persistent hypotension, hyper-or hypothermia, leukocytosis or leukopenia, and often thrombocytopenia (14). It was implied that the circulation system could be the key step for the ignition of a cytokine storm, promoting an inflammation from a local one to a severe systemic illness (15).

Coagulopathy in patients with COVID-19 has been reported with a high level of D-dimer (16). D-dimer, a degradation product of cross-linked fibrin indicating thrombosis, is widely used as an indicator of global activation of hemostasis and fibrinolysis. The neutrophil to lymphocyte ratio (N/L ratio) has been used as a clinical liquid biopsy marker for systemic inflammatory status in various disease types for many years (17). A correlation between the serum levels of interleukin-6 (IL-6) and the severity of COVID-19 symptoms has been reported (18, 19). The anti-IL-6 antibody tocilizumab is actively being tested in clinical trials by Xu et al. (19); the anti-IL-6 receptor antibody was also expanded in clinical trials for COVID-19 patients by Regeneron and Sanofi. The aim of this study was to observe the association of coagulopathy (D-dimer) with cytokines [i.e., neutrophil-to-lymphocyte ratio (NLR), IL-6, and C-reactive protein (CRP)] and CT imaging in COVID-19-infected patients.

## Methods

### Patient Selection

Patients were recruited from an in-patient unit of the First Affiliated Hospital of Bengbu Medical College. Sixty-four patients with a diagnosis of COVID-19 were included in the study. All the 64 patients were clinically evaluated and classified into three groups of mild, moderate, and severe condition with COVID-19 infection according to the Chinese Clinical Guidance for COVID-19 Pneumonia Diagnosis and Treatment (7th edition). There were 32 patients, including 15 males and 17 females, with a mean age of 54 years (range 33–73) in the mild group, 23 patients including 13 males, and 10 females with a mean age of 57 years (range 21–83) in the moderate group, and nine patients including eight males and one female, with a mean age of 61 years (range 47–81) in the severe group. Of the nine patients in the severe group, five died during a follow-up of the study. The ethical committee of the First Affiliated Hospital of Bengbu Medical College approved this study; the approval number is BYYFY-2020KY03.

The 64 COVID-19-positive patients were confirmed to have a viral load by nucleic acid real-time RT-PCR test (commercial kit specific for 2019-nCoV, DaAn Gene Co., Ltd., Guangzhou, China) conducted at least twice.

### Laboratory Data Collection

Complete blood count was tested on a blood analysis platform (XE-5000, Sysmex), with white blood cell count and classification using semi-flow fluorescent staining technology. NLR was calculated from the number of neutrophils and lymphocytes. D-dimer was measured by immune turbidimetry on an automated coagulation system (CS-5100, Sysmex). CRP was measured by immune turbidimetry on an automated biochemistry analysis platform (cobas 8000, Roche). IL-6 was tested with electrical chemical immune analysis technology (cobas e601, Roche). Procalcitonin was measured with a fluorescence immunochromatographic assay (QT-200, Wondfo).

### Follow-Up

After admission, the patients were routinely monitored for the laboratory results of routine blood test, CRP, and CT imaging when medical treatment was necessary. Follow-up occurred twice on the first month after discharge and then monthly for an additional 3 months.

### Statistics

The cutoff value of NLR was calculated based on the maximum Youden index. *T*-tests or chi-square tests were used to compare differences between two groups. ANOVA tests were used to compare the differences among mild, moderate, and severe groups. Correlations were analyzed with either Pearson or Spearman correlation coefficient. Analyses were performed using SPSS 22.0 statistical package (SPSS, Inc., Chicago, IL, USA) and R version 3.6.2. *P* < 0.05 was considered as statistically significant.

## Results

### D-dimer Correlates With NLR and IL-6 in COVID-19-Infected Patients

As previously reported, D-dimer levels over 1 μg/L at admission predicted an 18-fold increase in odds of mortality (12). However, the underlying mechanisms are unknown. NLR was widely reported as a biomarker in various types of diseases, including COVID-19 pneumonia. The neutrophil-to-lymphocyte ratio is believed to able to predict the immune status of patients. However, whether it is related with a coagulant system is unknown.

**Figure 1:**
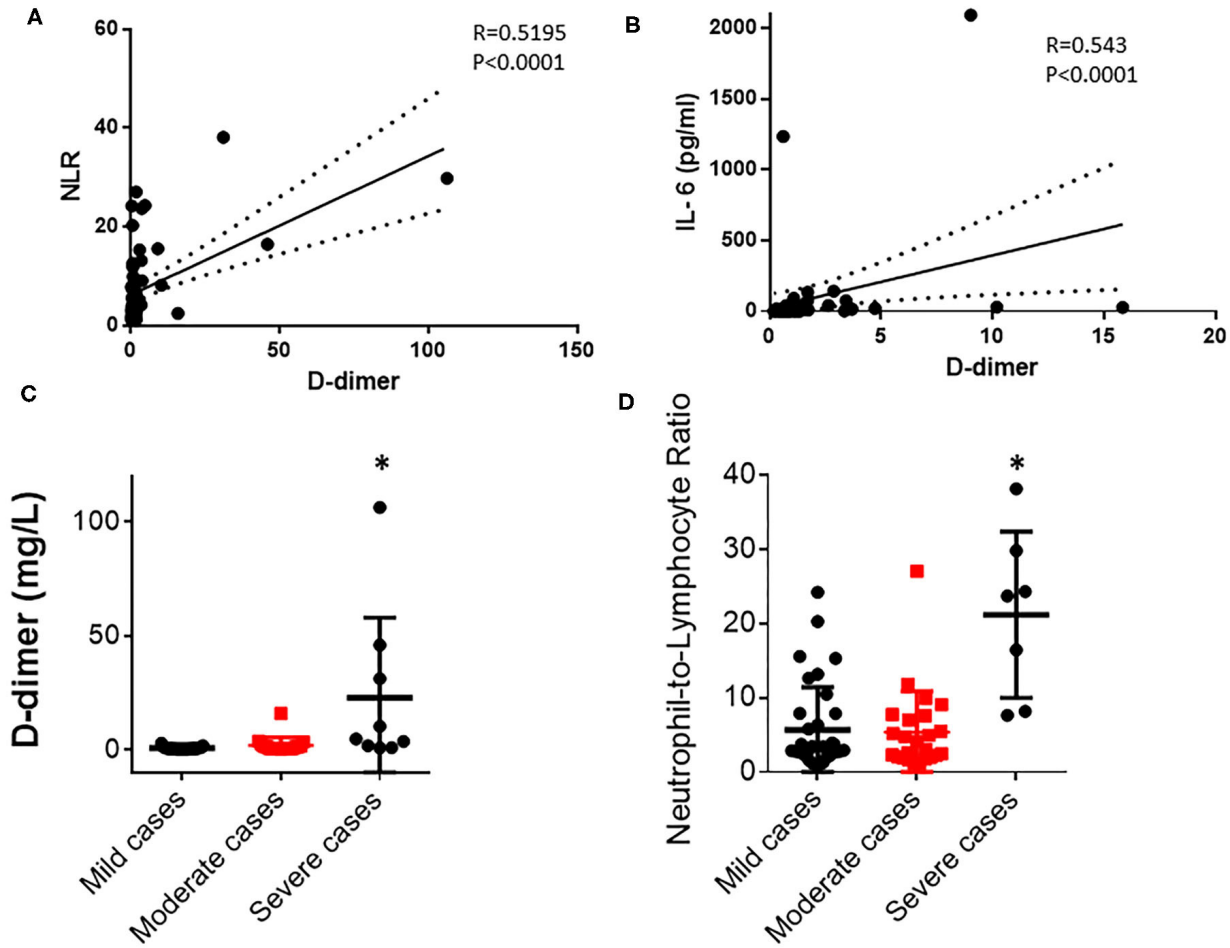
D-dimer correlates with neutrophil-to-lymphocyte ratio (NLR) and IL-6 in COVID-19-infected patients. **(A)** D-dimer level correlates with NLR in all 69 COVID-19 patients (Pearson correlation, *R* = 0.5195, *p* < 0.0001). **(B)** D-dimer level correlates with IL-6 level in the serum of all 69 COVID-19 patients (Spearman correlation, *R* = 0.543, *p* < 0.0001). **(C)** Comparison of D-dimer levels in mild, moderate, and severe groups (*p* = 0.0002). **(D)** Comparison of NLR in mild, moderate, and severe groups (*p* < 0.001). *p < 0.05.

To investigate how D-dimer contributes to the disease progression of a COVID-19 infection, we observed that the D-dimer level exhibited moderate correlations with NLR (*R* = 0.5195, *p* < 0.001; Figure 1A; Table 1) and IL-6 (*R* = 0.543, *p* < 0.0001; Figure 1B, Table 1) in COVID-19 infected patients. These data suggest that a coagulant system is highly likely to correlate with the immune status of COVID-19-infected patients. There are no differences in either D-dimer or NLR between the mild and the moderate groups (Figures 1C,D; Table 1). As expected, D-dimer is significantly higher in the severe group than the mild and the moderate groups (Figure 1C; Table 1) at baseline. Similarly, NLR is significantly higher in severe cases than in less severe cases (Figure 1D). Thus, these data imply a clinical link of coagulopathy with the immune status of COVID-19 patients.

**Table 1:** Baseline characteristics of Laboratory findings of patients with COVID-19.

**Characteristic**	**Normal Range**	**Mild cases** **(*n* = 32)**	**Moderate cases** **(*n* = 23)**	**Severe cases** **(*n* = 9)**	***P-*value**
M/F		M15/F17	M13/F10	M8/F1	
AGE		54 (33–73)	57 (21–83)	61 (47–81)	0.0736
GLU	3.9–6.1 mmol/L	6.2 (5.71–6.7)	8.45 (7.09–9.82)	9.78 (5.61–13.96)	<0.0001
LDL	1.07–3.3 mmol/L	2.30 (2.03–2.58)	2.29 (2.01–2.57)	1.52 (1.19–1.85)	0.0094
D-Dimer	0–0.55 mg/L	0.65 (0.42–0.87)	1.91 (0.3–3.51)	22.79 (−4.14–49.72)	0.0002
NLR		4.58 (2.64–6.52)	5.14 (3.6–6.69)	16.05 (6.39–25.72)	<0.0001
CRP	0–6 mg/L	38.05 (18.5–57.59)	58.72 (33.08–84.37)	147.87 (95.95–199.8)	<0.0001
PCT	<0.50 ng/mL	0.18 (0.12–0.23)	0.14 (0.12–0.17)	5.99 (−6.6–18.58)	0.0385
IL-6	<7 pg/mL	12.3 (2.20–22.39)	18.87 (8.27–29.39)	55.49 (−28–139)	0.0197
LY^#^	(1.1–3.2) * 10^9^/L	1.34 (1.14–1.55)	1.12 (0.86–1.38)	0.64 (0.33–0.96)	0.0056
MCH	27–34 pg	31.43 (30.71–32.15)	30.41 (29.36–31.46)	30.89 (29.21–32.57)	0.2394
MCHC	316–354 g/L	351.25 (345.6–356.9)	350.91 (342.6–359.2)	346.44 (337.5–355.4)	0.3088
MCV	82–100 fL	89.53 (87.91–91.14)	86.69 (84.52–88.86)	89.23 (83.89–94.58)	0.117
MPV	5.0–11.0 fL	9.525 (8.94–10.11)	9.38 (8.76–10)	9.54 (8.2–10.88)	0.9342
NEUT^#^	(1.8–6.3) * 10^9^/L	4.96 (3.22–6.69)	4.29 (3.61–4.96)	8.16 (4.8–11.52)	0.0438
PLT	(125–350) * 10^9^/L	247.16 (212.9–281.4)	269.13 (225–313.2)	172.11 (105.8–238.4)	0.0439
RDW-CV	11–15%	12.72 (12.5–12.94)	12.81 (12.5–13.12)	13.12 (12.2–14.04)	0.3739
RDW-SD	37–54%	40.93 (40.04–41.83)	39.83 (38.84–40.83)	41.82 (38.78–44.87)	0.1277
RET^#^	(0.024–0.084) * 10^12^/L	0.04 (0.029–0.045)	0.034 (0.027–0.04)	0.028 (0.019–0.036)	0.4071
WBC	(3.5–9.5) * 10^9^/L	6.92 (5.18–8.67)	5.89 (5.08–6.7)	9.17 (5.64–12.69)	0.1213
A/G	1.2–2.4	1.5 (1.36–1.64)	1.44 (1.24–1.64)	1.27 (1.01–1.52)	0.3197
AG	8–16 mmol/L	13.06 (11.71–14.4)	13.2 (11.41–14.98)	15.32 (11.07–19.57)	0.3285
ALB	40–55 g/L	39.13 (37.85–40.42)	37.12 (35.5–38.74)	33.54 (30.09–37)	0.0008
ALP	45–125 U/L	44.75 (38.69–50.81)	45.13 (39.07–51.19)	64.56 (44.17–84.94)	0.0109
ALT	9–60 U/L	26.25 (17.66–34.84)	35.65 (16.68–54.62)	281 (−320.9–882.9)	0.0574
APOA	0.9–1.6 g/L	0.93 (0.86–1.01)	0.76 (0.7–0.82)	0.61 (0.56–0.66)	<0.0001
APOB	0.6–1.1 g/L	0.78 (0.7–0.86)	0.76 (0.67–0.86)	0.55 (0.43–0.66)	0.017
AST	15–45 U/L	27.19 (22.01–32.36)	42.57 (22.88–62.25)	753.1 (−898.3–2405)	0.0434
CA^2+^	2.11–2.52 mmol/L	2.14 (2.1–2.18)	2.13 (2.06–2.2)	1.94 (1.86–2.02)	0.0005
LDH	125–250 U/L	286.97 (237.5–336.5)	322.96 (239.7–406.2)	1,040.56 (84.01–1997)	0.0004
sdLDL	0.27–1.44 mmol/L	0.80 (0.69–0.90)	0.75 (0.64–0.85)	0.39 (0.27–0.51)	0.0004

# *Absolute number*.

### D-dimer Rises Earlier Than IL-6 and NLR Flare/Cytokine Storm During Infection

**Figure 2:**
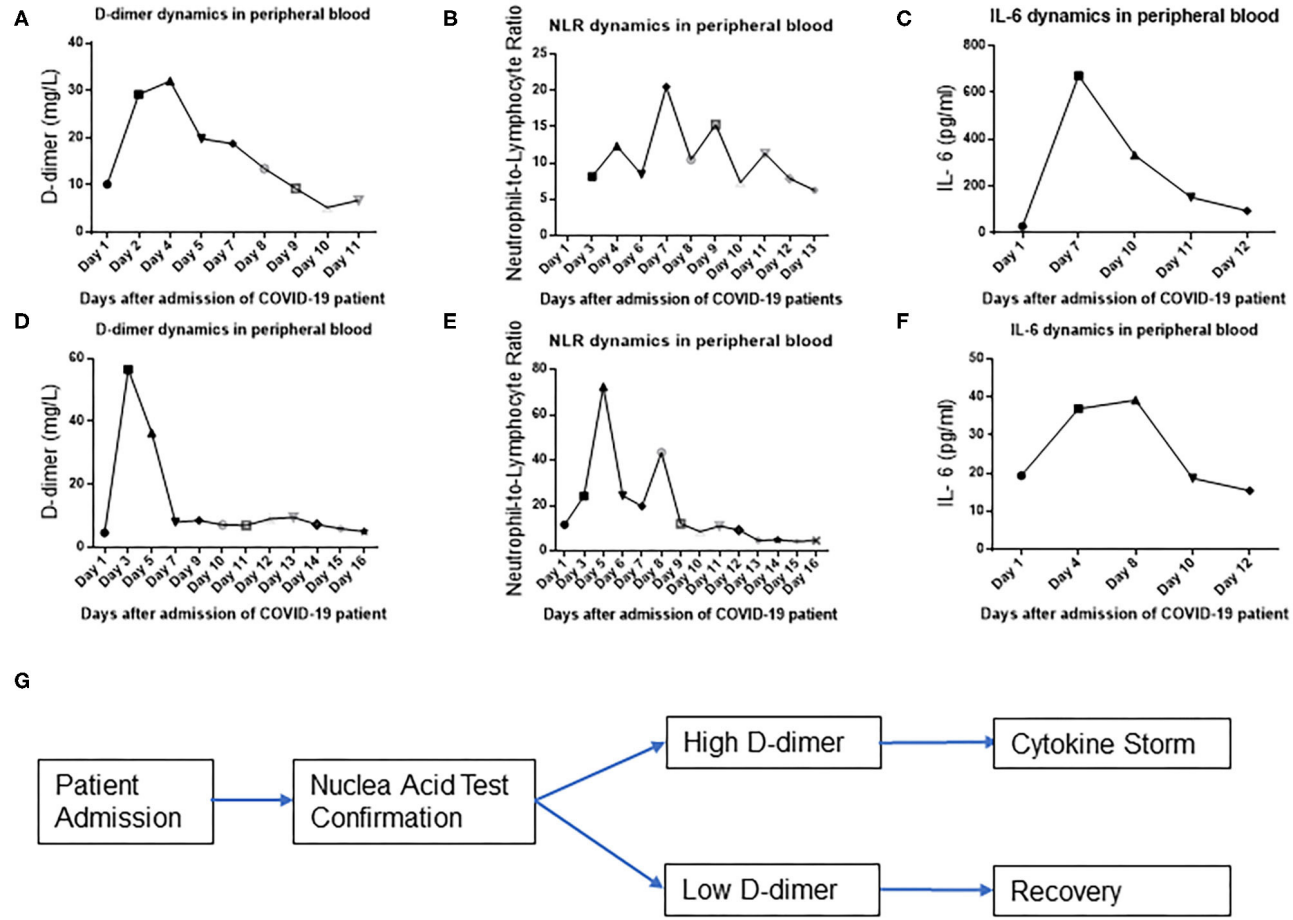
D-dimer precedes IL-6 surge in a severe group of COVID-19-infected patients. D-dimer **(A)**, neutrophil-to-lymphocyte ratio (NLR) **(B)**, and IL-6 **(C)** temporal dynamics of representative patient 1 in peripheral blood. D-dimer **(D)**, NLR **(E)**, and IL-6 **(F)** temporal dynamics of representative patient 2 in peripheral blood. **(G)** Schematic flow diagram of the results.

The abrupt emergence of a cytokine storm in COVID-19-infected patients is believed to be the most dangerous reason of a patient's death. However, the reason for an unexpected cytokine storm is unknown. The tissue and blood vessel damage could be a trigger of an abrupt cytokine storm. To test this hypothesis, we examined the timing and the temporal course of D dimer, NLR, and IL-6 level evolution in peripheral blood during COVID-19 infection in an individual patient with a severe condition. Surprisingly, D-dimer level surged at day 2 after admission (Figure 2A), whereas NLR (Figure 2B) and IL-6 (Figure 2C) levels started to surge at day 7 after admission in one patient with a severe illness condition. These findings demonstrate that the D-dimer level rises earlier than IL-6 glare/cytokine storm along with COVID-19 disease progression (Figures 2A–C). To look for more evidences of this temporal course of D-dimer- and IL-6-represented immune activations, we found that D-dimer rose at day 2 (Figure 2D) and NLR and IL-6 surged at around day 5 in another patient with a severe condition (Figures 2E,F), which suggest that D-dimer-associated tissue coagulopathy might predispose IL-6 production, NLR, and rapid immune overactivation along with COVID-19 disease progression (Figure 2G).

### D-dimer Correlates With CT Imaging

**Figure 3:**
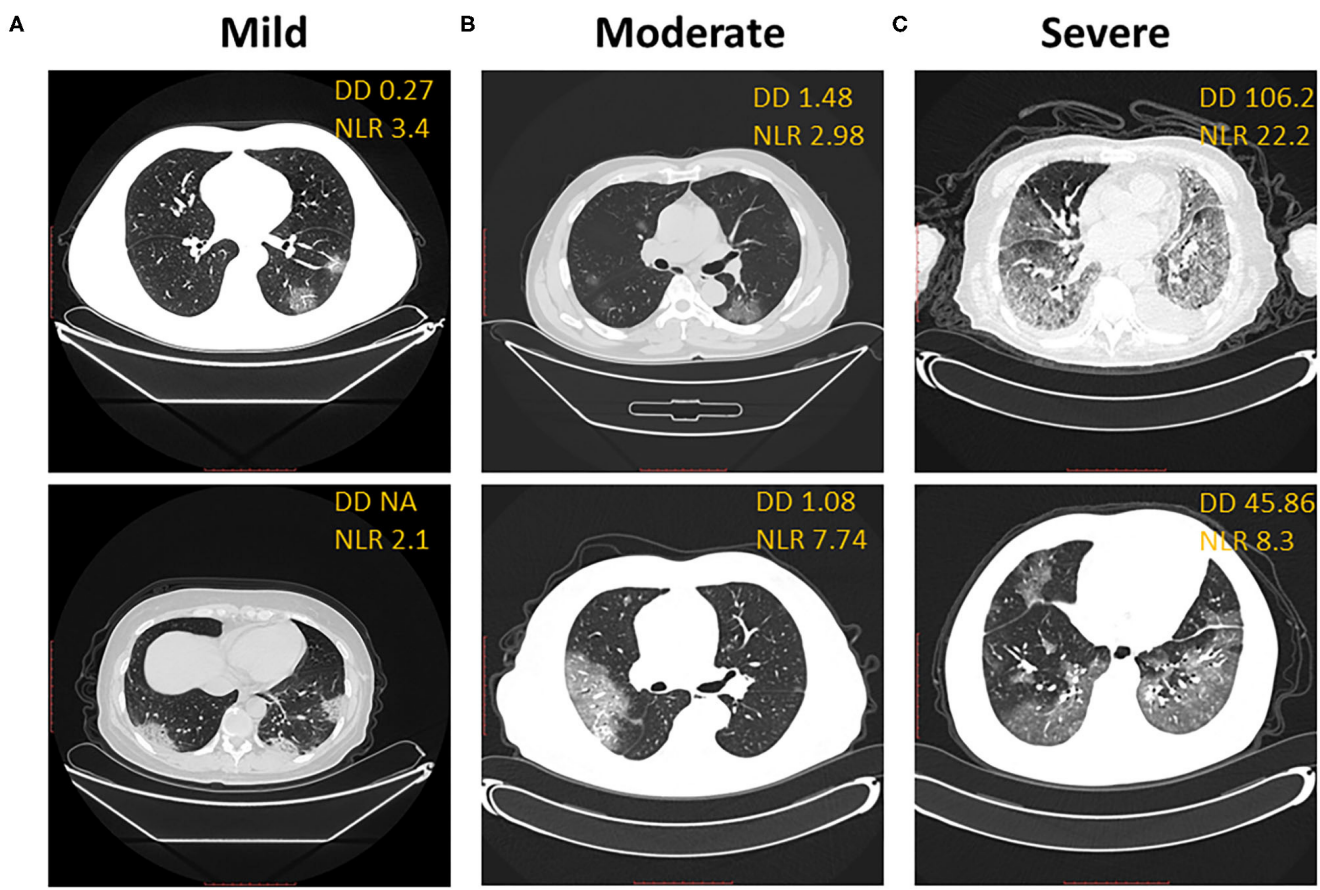
D-dimer correlates with CT imaging. Correlation of representative CT imaging with D-dimer and neutrophil-to-lymphocyte ratio in mild **(A)**, moderate **(B)**, and severe **(C)** groups of COVID-19 patients. **(A)** Ground-glass opacity in the bottom segment of the left lung, blood vessel-like. **(B)** Ground glass shadow expansion and consolidation in bilateral lung. **(C)** Overwhelming ground glass shadow distribution, bilateral patchy shadowing, and enlarged blood vessel.

CT imaging of the lung can reveal the severity of COVID-19 clinical symptoms and is one of the alternative clinical criteria to diagnose a COVID-19 infection. In our cohort of clinical data, the D-dimer level correlated with the ground-glass area of CT imaging and the severity of COVID-19 clinical symptoms at baseline when we compared the D-dimer level with CT imaging in the mild group (Figure 3A), the moderate group (Figure 3B), and the severe group (Figure 3C). The D-dimer level correlated with an increased NLR level from mild to moderate to severe patients. The D-dimer level also correlated with the ground-glass area patterns of CT imaging in patients with mild, moderate, and severe condition, respectively (Figures 3A–C).

## Discussion

To our knowledge, we are the first to observe that coagulopathy might act as the prodrome of a cytokine storm in COVID-19-infected patients. Coagulopathy appeared around a few days in advance of a cytokine storm. We also observed moderate correlations of D-dimer with NLR, IL-6 levels, and CT imaging of the lungs in COVID-19-infected patients. D-dimer was reported to correlate with proinflammatory cytokine levels and outcomes in critically ill patients (20). Our data, combined with the results of a previous study (20), might further advance our knowledge of the correlation of coagulopathy and cytokines in human diseases. The D-dimer surge, which is more sensitive than measuring cytokines, might be used to predict a cytokine storm in COVID-19-infected patients. This study indicated the critical clinical value of coagulopathy monitoring and the early requirement of an anti-coagulant therapy to prevent a cytokine storm in COVID-19-infected patients.

A cytokine storm has been observed and considered as the reason of death for COVID-19-infected patients (4, 5). A cytokine storm starts locally in the lungs and gets activated in the systemic circulation; patients must reverse this immune system overactivation (6). It implied that the circulation system is the key step for the ignition of a cytokine storm and the spread of inflammation from local to systemic (7). We reported the blood-system-derived cytokine storm in COVID-19 patients. Considering why the blood system is potent to activate a cytokine storm, if we link it with the basics of immunology, the principle could be antigen dependence—the sudden release of a tremendous amount of antigen provides the power to expand the inflammation into the whole body, systemically activating the immune system and releasing cytokines.

There might be a potential relationship between coagulopathy and neoantigen supply. The systemic immune illness of a cytokine surge requires a rapid mobilization of the human immune system. The toolbox for efficient immune system mobilization in COVID-19 patients remains a mystery. We speculate that coagulopathy might efficiently generate a lot of neoantigens in patients, which helps to efficiently mobilize the human body to over-produce cytokines.

The limitations of our study should be acknowledged. A small sample size from one hospital and the results from this population may not generalize to other populations. A large sample size and more diverse samples are needed to confirm the results. Future studies should analyze more COVID-19 patients from multiple clinical sites and confirm the findings among COVID-19 patients in different conditions, such as relative to age and cardiac risk factors.

In summary, we observed moderate correlations of D-dimer with NLR and IL-6 levels. These findings implicate further studies of early anti-coagulant treatment with cytokine storm in COVID-19 patients and the possibility of preventing deleterious cytokine damage in patients. This study, combined with previous observations of coagulopathy in COVID-19-infected patients (16, 21–23), will help the medical field to develop an effective clinical strategy. Anti-coagulant treatment could represent a novel preventive treatment strategy to block a severe clinical cytokine storm in COVID-19 patients with moderate or mild condition.

## Data Availability Statement

The original contributions generated for the study are included in the article/supplementary material, further inquiries can be directed to the corresponding author/s.

## Ethics Statement

The studies involving human participants were reviewed and approved by ethics committee of first affiliated hospital, Bengbu Medical College. Written informed consent for participation was not required for this study in accordance with the national legislation and the institutional requirements.

## Author Contributions

HG, WL, JL (6th Author), FL, ZZ, JL (11th Author), YZ, JG, GL, and LW collected the data. HG, YS, and JL (11th Author) analyzed the data, processed statistics, wrote the manuscript, and revised the manuscript. HG, JL (11th Author), and FW supervised the study. All authors contributed to the article and approved submission.

## Conflict of Interest

The authors declare that the research was conducted in the absence of any commercial or financial relationships that could be construed as a potential conflict of interest.
